# Characterizing the genetic differences between two distinct migrant groups from Indo-European and Dravidian speaking populations in India

**DOI:** 10.1186/1471-2156-15-86

**Published:** 2014-07-22

**Authors:** Mohammad Ali, Xuanyao Liu, Esakimuthu Nisha Pillai, Peng Chen, Chiea-Chuen Khor, Rick Twee-Hee Ong, Yik-Ying Teo

**Affiliations:** 1 Life Sciences Institute, National University of Singapore, Singapore, Singapore; 2 Saw Swee Hock School of Public Health, National University of Singapore, Singapore, Singapore; 3 NUS Graduate School for Integrative Science and Engineering, National University of Singapore, Singapore, Singapore; 4 Genome Institute of Singapore, Agency for Science, Technology and Research, Singapore, Singapore; 5 Department of Statistics and Applied Probability, National University of Singapore, Singapore, Singapore; 6 Department of Statistics and Applied Probability, Faculty of Science, National University of Singapore, Blk S16, Level 7, 6 Science Drive 2, Singapore 117546, Singapore

**Keywords:** Positive selection, Long haplotype, Population diversity

## Abstract

**Background:**

India is home to many ethnically and linguistically diverse populations. It is hypothesized that history of invasions by people from Persia and Central Asia, who are referred as Aryans in Hindu Holy Scriptures, had a defining role in shaping the Indian population canvas. A shift in spoken languages from Dravidian languages to Indo-European languages around 1500 B.C. is central to the Aryan Invasion Theory. Here we investigate the genetic differences between two sub-populations of India consisting of: (1) The Indo-European language speaking Gujarati Indians with genome-wide data from the International HapMap Project; and (2) the Dravidian language speaking Tamil Indians with genome-wide data from the Singapore Genome Variation Project.

**Results:**

We implemented three population genetics measures to identify genomic regions that are significantly differentiated between the two Indian populations originating from the north and south of India. These measures singled out genomic regions with: (i) SNPs exhibiting significant variation in allele frequencies in the two Indian populations; and (ii) differential signals of positive natural selection as quantified by the integrated haplotype score (iHS) and cross-population extended haplotype homozygosity (XP-EHH). One of the regions that emerged spans the *SLC24A5* gene that has been functionally shown to affect skin pigmentation, with a higher degree of genetic sharing between Gujarati Indians and Europeans.

**Conclusions:**

Our finding points to a gene-flow from Europe to north India that provides an explanation for the lighter skin tones present in North Indians in comparison to South Indians.

## Background

India is one of the most populous countries and spans a significant amount of land area in south Asia. As a country, India is ethnically and linguistically diverse, and several studies have studied the genetic aspect of this diversity in Indian populations [1-10]. A strict caste system has existed in Indian societies for centuries, and this has limited inter-caste gene flow. The country also possesses two major ethno-linguistic groups: (i) the Indo-Aryan language speaking groups that are primarily present in north India; and (ii) the Dravidian language speaking groups that are predominantly in south India. Historical evidence suggests that prior to 1500BC, Dravidian languages were present throughout India, but there was a documented shift in the prevalence of the spoken languages towards Indo-Aryan languages after 1500BC [11]. This change in the dominant spoken languages in India is central to the theory where the Aryans, who traced their origins from Iran and Central Asia, invaded India and settled in the sub-continent. Strong archaeological evidence suggests the presence of an ancient civilization along the banks of the Indus river valley, an area located in the north-western region of the Indian subcontinent, and the subsequent disappearance of this civilization has been postulated by historians and anthropologists to be attributed to the Aryan invasion [3].

**Figure 1:**
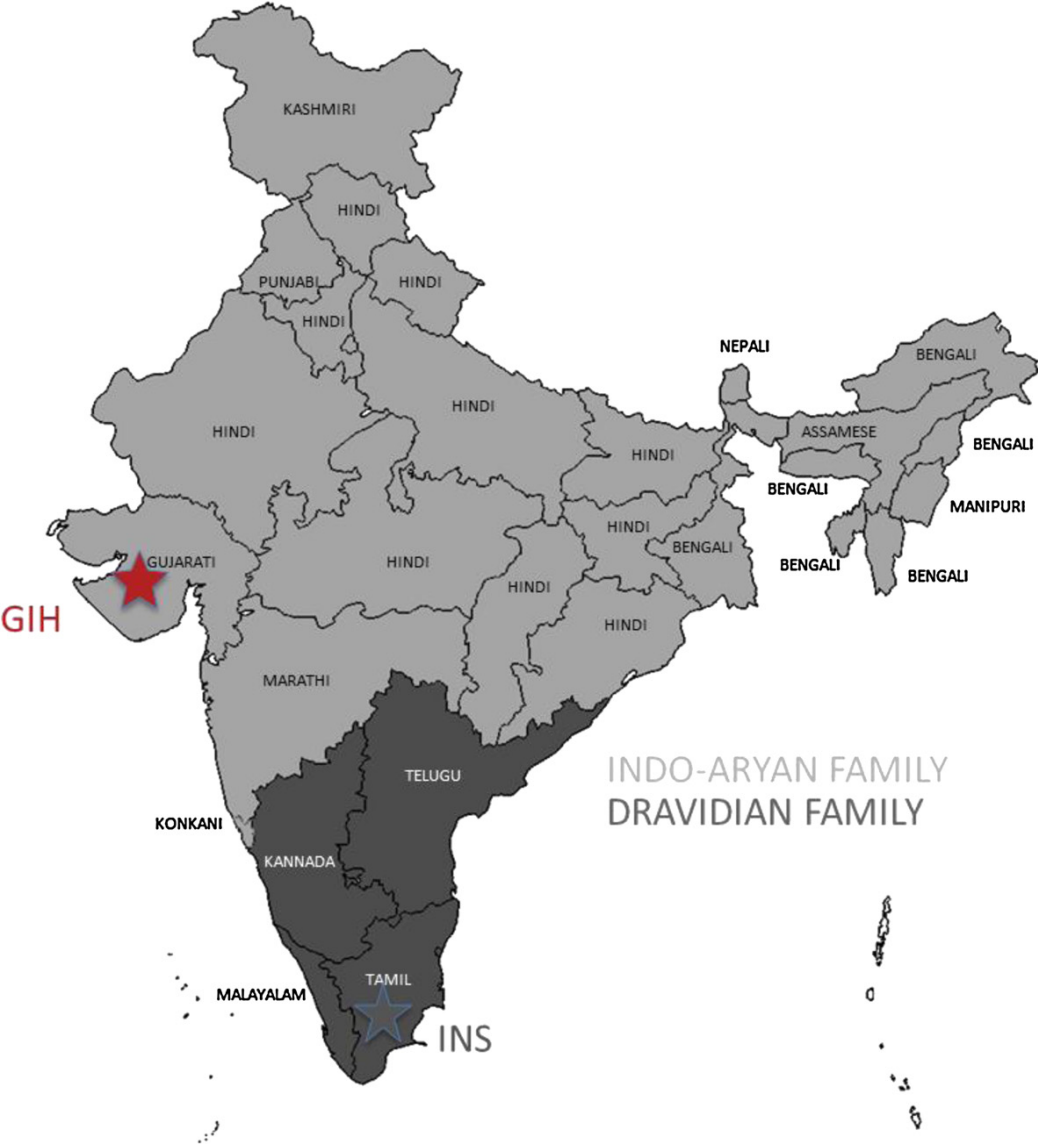
**Geography and language distribution of India.** In this map of India, all the states have been shaded according to the languages predominantly spoken in those states. The two broad language families are: (i) Dravidian (darker shade); and (ii) Indo-Aryan (lighter shade). There is a clear north–south divide, with northern states predominantly speaking Indo-Aryan languages such as Hindi, Marathi, Oriya, Punjabi and Gujarati; while southern states predominantly speak Dravidian languages such as Tamil, Malayalam, Kannada and Telugu. The two groups of samples used in this report trace their ancestries to different ethno-linguistic groups found at different geographical locations. The Houston Gujaratis (GIH) trace their ancestry to the Gujarati-speaking state of Gujarat (red star), while the Singapore Indians (INS) trace their ancestry predominantly to Tamil Nadu, a Dravidian-language speaking state in the south (grey star).

The presence of the caste system along with two major distinct language families has altered the mating pattern in Indian societies, and this has magnified the diversity of the gene pools that are present in the Indian subcontinent. One of the most apparent differences between north Indians and south Indians is in skin complexion, where north Indians are much fairer compared to the darker south Indians [12]. In this paper, we investigate genome-wide single nucleotide polymorphism (SNP) data from two Indian subpopulations: (i) the Gujaratis from Houston in Phase 3 of the International HapMap Project [13]; and (ii) the south Asian Indians from Singapore in the Singapore Genome Variation Project [14] (Figure 1). The Gujarati samples trace their roots to the Western State of Gujarat where the native language of the ethnic subgroup is classified as Indo-Aryan. The Indian population in Singapore predominantly descended from immigrants from Dravidian-speaking states of Kerala, Karnataka and Tamil Nadu, and thus most Singapore Indians can be regarded as representatives of the broader category of Dravidian-speaking south Indians [15].

Reich and colleagues [1] were amongst the first to investigate in detail the complex genetic canvas of India. They surveyed 132 Indians from 25 ethno-linguistically and socially distinctive groups across 560,123 SNPs, and reported the genetic substructures that are present across the Indian populations. However, the sample size of less than 10 for each sub-group does not provide sufficient resolution to confidently investigate genomic variability such as allele frequency differences and natural selection among different Indian subpopulations. For our analysis, we had 83 samples that trace their ancestry from the aforementioned states in south India, and 85 samples from individuals with lineage from the state of Gujarat. Furthermore, for our samples we had data from around 1.4 million (1,389,511) and 1.6 million (1,583,455) SNPs from the Indo-European language speakers and Dravidian language speaker groups respectively. The larger number of samples coupled with higher SNP densities across the genome presents the opportunity to interrogate the genome for regions that are substantially different between the northern Indians and the southern Indians.

Here, we use three population genetics metrics for quantifying genomic diversity between the north Indians and south Indians: (i) the Wright’s F_ST_ provides a measure of the variation in allele frequencies between populations [16]; (ii) the integrated haplotype score (iHS) provides a measure of the evidence for positive selection [17], which we subsequently search for genomic regions where there are significant differences in the iHS evidence in north Indians and south Indians; and (iii) the cross-population extended haplotype homozygosity (XP-EHH) score that investigates differential evidence of long haplotypes between two populations [18]. These metrics have previously been used successfully to identify genomic regions that differ between north and south Han Chinese [19], and we now extend the use of these metrics to explore the genetic architecture of Indian subpopulations, as well as to investigate whether positive selection is able to explain the emergence of genetic differences between the two groups.

## Results

To evaluate the extent of genetic differences that exists between a north Indian population that predominantly speaks the Indo-Aryan language, and a south Indian Dravidian-language speaking population, we considered the 85 Gujarati samples residing in Houston, Texas, from Phase 3 of HapMap (GIH) and the 83 samples from Singapore that are predominantly Tamil Indians (INS) from the SGVP (INS). A total of 1,362,474 autosomal SNPs that are present in both databases and phased haplotypes from the two public resources were used. Our interrogation for genetic evidence of north–south differences focused on three aspects of population genetics: (i) searching for genomic stretches that contained an excess of SNPs where the allele frequencies are markedly different between GIH and INS as quantified by the F_ST_ metric; (ii) regions under pressure of positive selection in only one of the two populations as quantified by the iHS metric; (iii) regions where XP-EHH exhibited significant evidence of differential selection between GIH and INS. To minimize the chance of false positive findings, we require any regions that have been identified by any one of the three criteria to be validated in at least one of the remaining two criteria, although the validation criteria were less stringent (see Table 1).

**Table 1:** Discovery and validation criterion for differentiated genomic regions

**Criteria**	**Discovery criterion**	**Validation criterion**
**F**_**ST**_ Region with an over-representation of SNPs possessing high F_ST_ values relative to the genome-wide distribution of F_ST_ scores	Regional evidence in the top 0.1% of the genome-wide distribution, in which:	Discovered region should contain evidence found in the top 1% of the genome-wide distribution
	- Regions are defined by window sizes of 100 kb and 500 kb;	
	- Evidence is defined by the P-value of the exact Binomial test for the proportion of SNPs with F_ST_ in the top 1st percentile (100 kb) or 0.1st percentile (500 kb) respectively of the genome-wide distribution score	
**Differential iHS signals for GIH and INS**	At least one SNP with normalized iHS score in the top 0.19% of the genome-wide distribution in one population, but not present in the top 1% of the genome-wide distribution in the other population	At least one SNP in the discovered region should have an iHS score in the top 1% of the genome-wide distribution, but absent in the top 1% of genome-wide distribution of iHS scores in the second population
**XP-EHH between GIH and INS**	Normalized XP-EHH scores should lie in the top 0.01% of the genome-wide distribution	At least one SNP in the discovered region should lie in the top 0.5% of the genome-wide distribution of the normalized XP-EHH scores

A description of the population genetics metrics used to discover and validate genomic regions that are differentiated between the north Indian Gujarati population (GIH) and the south Indian Tamil population from Singapore (INS).

*Abbreviations:iHS* integrated haplotype score, *XP-EHH* cross-population extended haplotype homozygosity.

We investigated the extent of population structure between the GIH and INS samples with three approaches: (i) principal components analysis (PCA); (ii) Wright’s fixation index (F_ST_); and (iii) the program *structure* that aims to assign population membership of each individual to a pre-specified number of populations.

**Figure 2:**
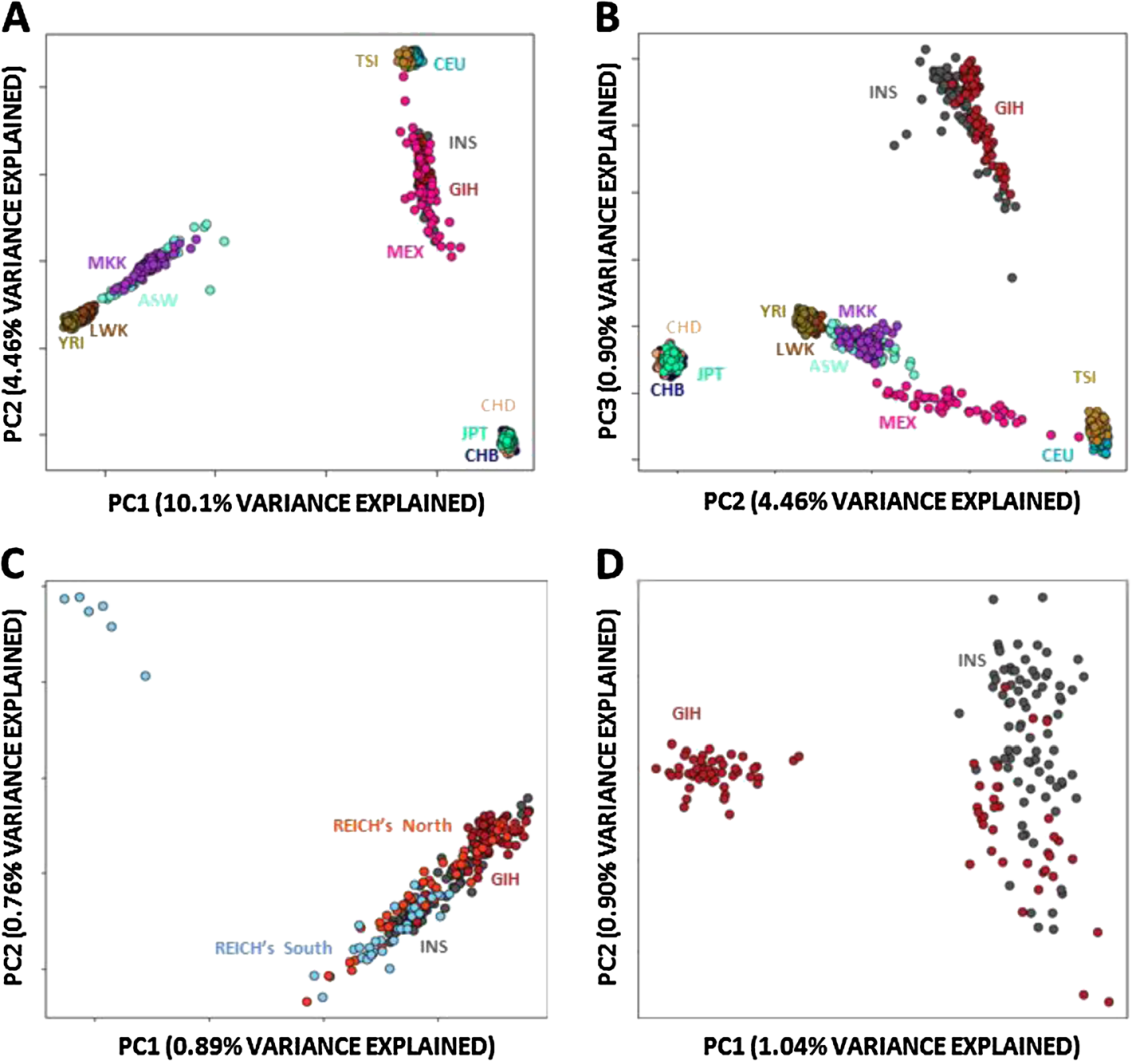
**Population structure of the Indians.** Principal components analyses of the two Indian populations (HapMap Gujarati: GIH, SGVP Tamil Indians: INS) with other global populations across 112,925 SNPs. **(A)** The first two principal components (PCs) of GIH and INS with the remaining ten populations from Phase 3 of the International HapMap Project. **(B)** Second and third PCs of the PCA of GIH and INS with the ten HapMap3 populations. **(C)** First two PCs from the analysis of GIH, INS and the 45 Indo-European speaking samples and 46 Dravidian-language speaking samples from the paper by Reich and colleagues. **(D)** First two PCs from the analysis with only GIH and INS samples.

Performing the PCA at a global scale where we compared GIH and INS with the remaining 10 populations in HapMap Phase 3, the GIH and INS samples were clustered together and were not immediately distinguishable with the first two principal components (Figure 2A), although a marginal separation between the two Indian populations was evident with the second and third principal components (Figure 2B). Comparing the two populations against the 132 samples from a survey of the population diversity of India by Reich and colleagues [1], we observed that GIH samples clustered closer to north Indian samples while INS samples were appropriately located with most of the south Indian samples (Figure 2C). An interesting pattern emerged from the PCA of only the GIH and INS samples (Figure 2D), where there were a group of 51 GIH samples that were more homogeneous among themselves and were clearly distinct from the INS samples; while the remaining 34 GIH samples were considerably more homogeneous to the INS samples.

We quantified the genetic distance between populations with the average F_ST_ calculated across 1,362,474 SNPs that are present in all the HapMap3 and INS populations. We observed that the genetic differentiation between the two Indian populations (average F_ST_ = 0.38%) were found to be larger than the distances between northern and southern Han Chinese populations in HapMap and SGVP respectively (CHB and CHS, average F_ST_ = 0.20% [14]), but was comparable to that observed between north-Western Europeans and the Toscans in Italy (CEU and TSI, average F_ST_ = 0.38%), and was less than the distances between any two African populations (LWK, MKK, YRI, average F_ST_ ≥ 0.62%).

**Figure 3:**
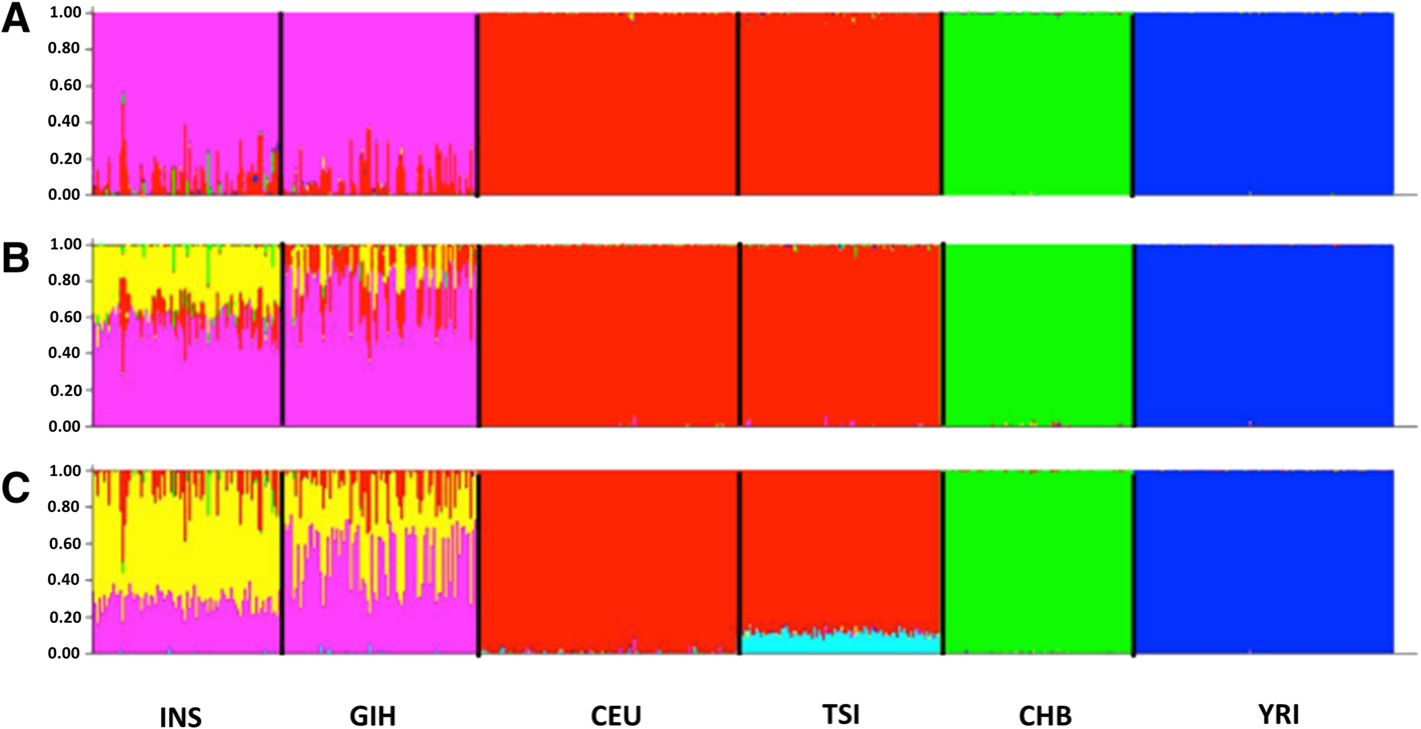
**Population structure analysis with STRUCTURE.** Analysis output from one of the five STRUCTURE runs with 10,000 randomly chosen autosomal SNPs for 85 Gujaratis (GIH), 83 Tamil Indians (INS) and another 399 individuals of north and western European ancestry (CEU), Toscans in Italy, Han Chinese in Beijing and Yoruba from the Ibadan region of Nigeria. Three separate analyses are performed for each set of 10,000 SNPs, where the number of subpopulations was set to four **(panel A)**, five **(panel B)** and six **(panel C)**. The analysis was performed with a burn-in of 10,000 iterations and for 20,000 samplings, where the posterior mean estimates across the 20,000 samplings were used to calculate the admixture proportion from the *K* populations for each individual.

The *structure* analyses were performed with the two Indian populations and four populations from another three ancestry groups (Europeans: CEU, TSI; East Asians: CHB; Africans: YRI) at three settings where the number of populations *K* was set to 4, 5 and 6. At *K* = 4, the Europeans, East Asian and African samples were assigned almost homogeneously to unique populations whereas the Indian samples, while clearly distinguishable from the other three ancestry groups, showed evidence of admixture with the Europeans (Figure 3A). The analysis at *K* = 5 further differentiated the two Indian populations, although it was evident there was a significant degree of admixture between the two Indian populations, and a small fraction of the Indian genomes mapped to the Europeans (Figure 3B). The findings about the two Indian populations were similar at *K* = 6, which essentially differentiated the north-Western Europeans (CEU) from the Italian Toscans (TSI) (Figure 3C).

The population structure analyses with PCA, F_ST_ and *structure* indicated the two Indian populations are genetically distinguishable, and this motivated further analyses to locate where the genetic differences are in the human genome. The availability of larger sample sizes in HapMap and SGVP allows for better inference of allele frequency differences, as well as for interrogating the genome for differential signatures of positive natural selection. We can thus search for genomic regions where there are substantial differences in the allele frequencies of the SNPs in these regions, and to investigate whether such differences are the consequence of different evolutionary pressure where positive selection is present in one population but not the other. Formally, a region is only identified if at least two of the following conditions are met: (i) the region corrected for nominal SNP density contains an excess of SNPs with significant differences in allele frequencies between GIH and INS; (ii) there are differential evidence from iHS such that one population exhibits evidence from iHS of positive selection while the other population does not; and (iii) there is evidence from XP-EHH of differential haplotype lengths between GIH and INS. The details of the discovery and validation criteria with these three metrics can be found in Table 1.

**Figure 4:**
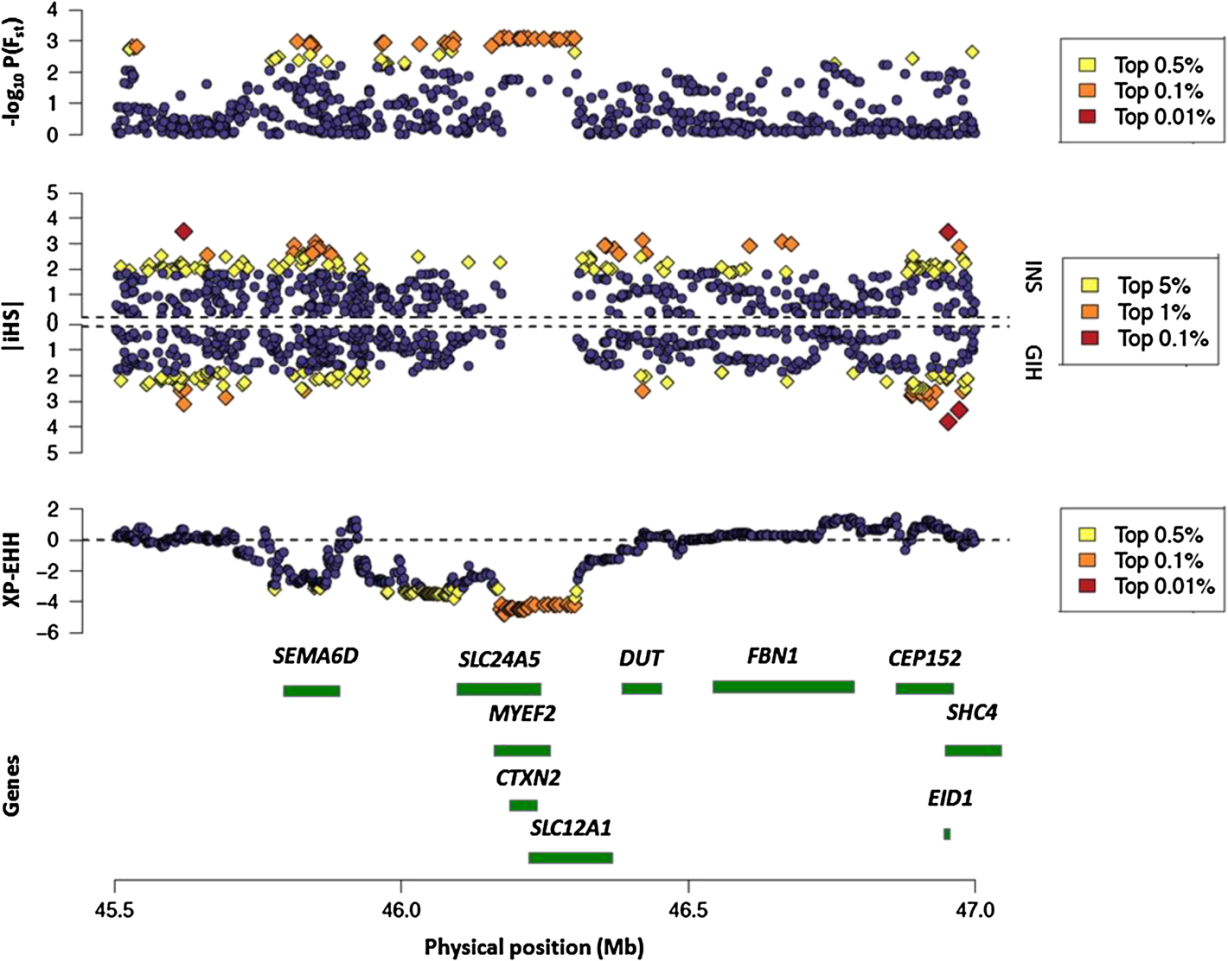
**Genetic differentiation between GIH and INS on chromosome 15.** Evidence of genetic differentiation between GIH and INS on chromosome 15 between 45.5 Mb and 47.0 Mb from three discovery mechanisms that look for considerable regional differences in SNP allele frequencies (as quantified by the F_ST_ metric) relative to the genome (top panel); differential evidence of positive selection from iHS in GIH and INS (middle panel); XP-EHH signals contrasting GIH and INS that are found in either tails of the genome-wide distribution (bottom panel). In all three panels, SNPs exhibiting extreme evidence relative to the genome are displayed in differently in yellow, orange and red according to the respective percentiles as illustrated in the three figure legends. Genes located within this region are identified according to NCBI hg18 (build 36) coordinates.

A total of eight regions were identified from our analyses, of which six regions encompassed at least one gene (see Table 2). One of these six regions is the region on chromosome 15 between 45.7 Mb and 46.2 Mb, which encapsulated four genes including the solute carrier family 24 member 5 (*SLC24A5*) gene that has been associated with skin pigmentation [20] (Figure 4). This region was found to exhibit regional differences in allele frequencies at the top 0.1% of the genome-wide distribution, along with XP-EHH signals found at the extreme 0.1th percentile, where the direction of the XP-EHH region corresponded with evidence of positive selection in GIH relative to INS. A genome-wide association study of skin pigmentation in a South Asian population identified the guanine allele for the index SNP rs1834640 in *SLC24A5* to be associated with lighter skin pigmentation, and this allele was present at a frequency of 4.7% in INS compared to 40.2% in GIH (F_ST_ = 18.1%), indicating that the differential evidence in this region concurs with the significant difference in the frequency of an allele that has been linked to skin pigmentation.

**Table 2:** Significantly differentiated regions

**Discovery mechanism**	**Chr**	**Start (Mb)**	**End (Mb)**	**FST (window size)**	**XP-EHH (direction**^**a**^**)**	**iHS (INS)**	**iHS (GIH)**	**Genes**
**F**_**ST**_	4	17.0	17.5	Top 0.1% (500 kb)	No evidence	No evidence	Top 1%	*QDPR, FAM184B, CLRN2, DCAF16, LAP3, MED28*
	5	105.4	105.7	Top 0.1% (100 kb)	Top 0.5% (negative)	No evidence	No evidence	*-*
	8	85.0	85.6	Top 0.1% (500 kb)	Top 0.5% (negative)	No evidence	No evidence	*RALYL*
	15	45.7	46.2	Top 0.1% (500 kb)	Top 0.5% (negative)	No evidence	No evidence	*SLC24A5, MYEF2, CTXN2, SLC12A1*
	17	41.3	41.5	Top 0.1% (100 kb)	Top 0.5% (positive)	No evidence	No evidence	*MAPT, STH, KIAA1267*
**iHS**	12	23.0	23.3	Top 1% (100 kb)	No evidence	No evidence	Top 0.1%	*-*
	12	58.3	58.6	No evidence	Top 0.5% (negative)	No evidence	Top 0.1%	*SLC16A7*
	12	80.3	80.6	Top 1% (100 kb)	No evidence	Top 0.1%	No evidence	*ACSS3, PPFIA2*

Regions identified and validated across the genome by different discovery mechanisms using three population genetics metrics calculated from the data for GIH and INS.

^a^Positive direction indicates evidence of positive selection in INS while negative direction indicates evidence of positive selection in GIH.

Another region that emerged with consistent evidence from regional F_ST_ and XP-EHH was found on chromosome 17 between 41.3 Mb and 41.5 Mb (Additional file 1: Figure S1) and encompassed three genes, two of which (*STH* and *KANSL1*) have previously been implicated with variation in intracranial volume [21] and the microtubule-associated protein tau (*MAPT*) gene has been consistently reported to be associated with Parkinson’s disease in Europeans [22-24]. The evidence from XP-EHH suggests the presence of positive selection at this locus in INS and not in GIH.

The remaining four regions encompassed genes that have not been reported for any phenotypic associations, but met our criteria where at least two of the three metrics were found at the extreme end of the respective genome-wide distributions. For example, the region on chromosome 4 between 17.0 Mb and 17.5 Mb was identified by the F_ST_ criterion and was further corroborated by evidence from iHS in the top 1% in GIH but not in INS (Additional file 1: Figure S2). This is similarly the case for the region identified on chromosome 8 between 85.0 Mb and 86.0 Mb by F_ST_, and where the region exhibited evidence of positive selection in GIH with XP-EHH (Additional file 1: Figure S3). Two regions on chromosome 12 at 58.3 Mb-58.6 Mb and 80.3 Mb-80.6 Mb exhibited differential evidence of positive selection according to iHS. In the former region that encompassed *SLC16A7*, an iHS signal at the top 0.1% of the distribution was present in GIH but there was no corresponding signal in INS even at a lower genome-wide significant threshold of 1% (Additional file 1: Figure S4). In the latter region which encompassed the *ACSS3* and *PPFIA2* genes, the iHS signals were present at the top 0.1% in INS but not at the top 1% in GIH (Additional file 1: Figure S5).

An extension to searching for differential evidence of positive selection in north and south Indians is to measure the relative degree of haplotype sharing between north Indians with Europeans, and with south Indians. We calculated the haplotype similarity score [25], a numerical metric bounded between 0 and 1 where a larger value indicates a greater degree of haplotype sharing, between GIH and TSI, and between GIH and INS. The primary interest here is to search for genomic regions where the haplotype similarity score is greater than 0.5 between one pair of populations while lower than 0.5 in the other pair, and this is meant to indicate which population the GIH haplotypes is more similar to. In our analysis where we divided each chromosome into non-overlapping windows of 100 kb each, there were 1,455 windows each of size 100 kb where GIH haplotypes were more similar to INS haplotypes, as compared to 679 windows where GIH haplotypes were more similar to TSI haplotypes. This indicated that there was still a greater degree of sharing between GIH and INS than with TSI, a finding that concurred with the results of the PCA and STRUCTURE analyses.

## Discussion

In this paper, we attempted a systematic, genome-wide search for regions showing significant evidence of differentiation between north and south Indians. To this end, we compared the genome-wide data from the two public databases of the International HapMap Project and the Singapore Genome Variation Project. The HapMap project surveyed 88 Gujarati Indians from Houston while the SGVP included 83 Indians with ancestry primarily from the south of India. We observed that the genetic distance between the two Indian groups was comparable to that between north-western Europeans and southern Europeans, but was further apart than northern and southern Han Chinese. The genetic dissimilarity between north and south Indians were discernible in the PCA and *structure* analyses, and eight genomic regions were identified in our analyses to exhibit significant evidence of genetic differentiation between the two groups of Indians.

In one of the eight regions lies the *SLC24A5* gene that has been functionally established to affect skin pigmentation in both humans and zebrafish [26]. The functional variant in this gene has also been proposed as an ancestry informative marker, as the variant allele is almost fixed in European populations and correlates with lighter skin pigmentation in admixed populations [27]. A genome-wide association study of skin pigmentation in south Asian populations similarly identified markers in this gene to differentiate between fairer and darker skin pigmentation [12]. Our discovery of this region is thus both exciting and reassuring, since this provides a well-established positive control in our analyses into the molecular genetics of the differences between north and south Indians.

**Figure 5:**
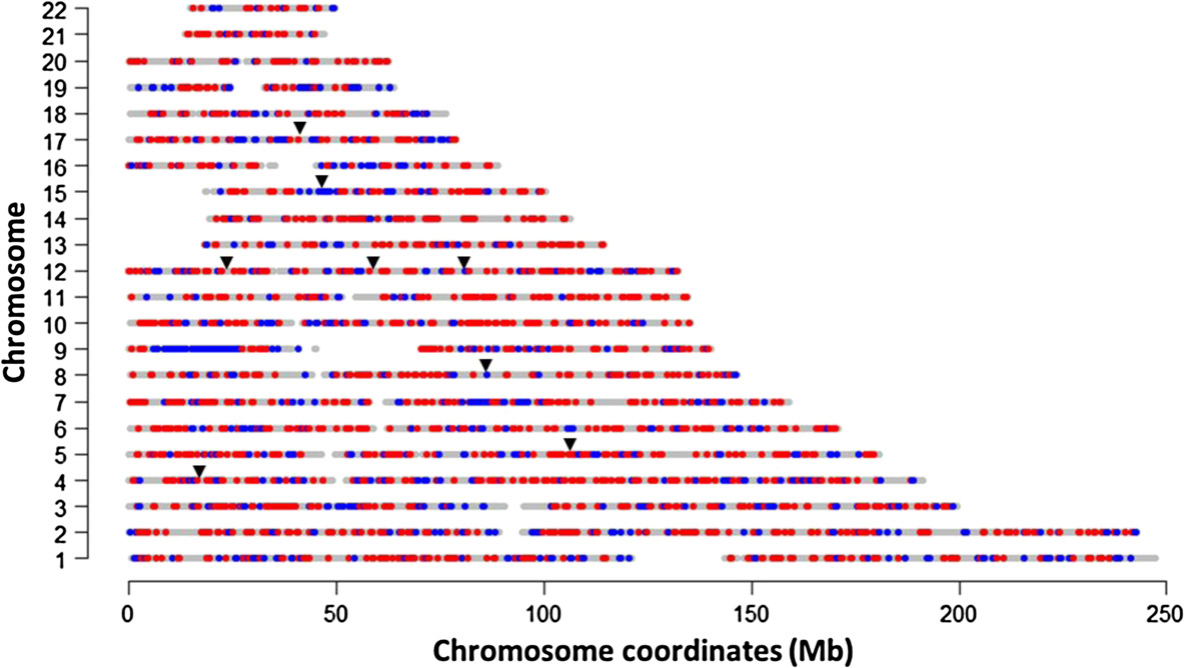
**Genomic similarity between north Indians to south Indians or south Europeans.** Chromosomal representation of the 22 autosomal chromosomes by whether the Gujarati Indian (GIH) haplotypes are more similar to the Italian Toscan (TSI) haplotypes, or whether the GIH haplotypes are more similar to the Singapore Tamil Indian (INS) haplotypes. Regions in grey indicate the assayed portions of each chromosome; a region is coloured in blue when the haplotype similarity between GIH and TSI is above 0.5 while the haplotype similarity between GIH and INS is less than 0.5; and a regions is coloured in red when the haplotype similarity between GIH and INS is greater than 0.5 while the haplotype similarity between GIH and TSI is less than 0.5. To obtain the haplotype similarity score for each non-overlapping window of 100 kb, the set of unique haplotypes window that are present with frequencies of at least 2% in each population is first collated. Then the haplotype similarity score is defined as the proportion of the haplotypes from the two populations that have been represented by these haplotypes. The metric is bounded between 0 and 1, with larger values indicating greater degree of haplotype sharing between the two populations. The black triangles indicate the positions of the eight regions identified by our three population genetics metrics.

The discovery that the region carrying *SLC24A5* is positively selected in north Gujarati Indians but not in south Tamil Indians may actually provide the first molecular evidence to support the belief of sexual selection, where members of most Indian societies have the tendency to prefer partners with fairer skin complexion [28]. Traditional upper castes from north India tend to be Indo-Aryan language speakers and are associated with fairer skin complexion, and there tended to be little vertical inter-caste marriages [28]. This would agree with previous reports of north-western Indians and those from upper castes across India having a greater degree of genetic similarity to that present in central and west Asia, as well as parts of Europe [1,3,29,30], despite these reports having relied on far lesser amount of data from chromosome Y or mitochondrial DNA. A particular chromosome Y haplogroup (U7) was previously reported to be present at higher frequencies in Gujarat and west Eurasia, but were almost non-existent in other parts of India [31]. Indeed in a recent report describing a novel methodology to locate and trace the origins of genomic signatures of positive selection, the selection signal present across *SLC24A5* in the Gujarati samples in the HapMap was reported to share the same origins as the selection signals present in north-western and southern Europeans [32]. Our analysis of haplotype similarity at the *SLC24A5* region between the Gujarati Indians also indicated greater degree of sharing with the southern Europeans than with the south Tamil Indians (Figure 5). A recent paper by Mallick and colleagues similarly reported evidence of positive selection at this gene with the use of sequence and genotyping data in separate cohorts from the North India, Pakistan, Central Asia and Middle East, although they observed concurring evidence as ours that selection was conspicuously absent in South India [33]. With the greater amount of data from a diverse panel of South Asian and West Eurasian populations, Mallick and colleagues similarly reported the monophyletic nature of the functional allele prescribing lighter skin pigmentation to exist on a distinct haplotype form that is common to both the South Asian and West Eurasian populations [33].

One striking omission in the differentiated regions is the Major Histocompatibility Complex (MHC) that has often been reported to be differentiated even between closely related populations. In all three metrics, we did not observe any significant evidence of genomic dissimilarity between the two populations at this region on chromosome 6. Although the three metrics are not strictly independent, they survey different features of the genomic architecture, from measuring differences at the allelic level (F_ST_) to comparing haplotype structures and the decay of haplotypes (XP-EHH and iHS). A priori, we expected the MHC to emerge as one of the differentiated regions, but there were no evidence even at the SNP-level to indicate that the allelic spectrum was significantly different between the two populations. This differed from the observations made in the Han Chinese, where segments of the MHC emerged as one of the differentiated regions between north and south Chinese [25].

A prominent feature of the PCA analysis was the grouping of 34 Gujarati Indians with the Singapore Tamil Indians. Our subsequent analyses did not exclude or partition these 34 GIH samples from the remaining 51 samples as we have sought to explore the genetic differences between two different ethno-linguistic groups that traced their ancestries from two different geographical regions of India. The Gujarati samples in our analyses have been defined by HapMap to be individuals of Gujarati descent, and we believed it will not be appropriate to redefine the ancestry or population labels of these samples, particularly since the PCA alone does not provide adequate evidence to ascertain that these 34 samples do not have a Gujarati ancestry. Instead, we believe this is exactly the form of genetic evidence to support and strengthen the belief that India is an ethnically and linguistically diverse country, where social customs have traditionally been governed by strict caste and religious systems, and where broad definitions of population groupings in India are likely to mask the complex sociological and genetic structures that are present in Indian societies. We thus advocate the collection of more detailed information with respect to caste and religion in future population genetics survey in India.

## Conclusion

To the best of our knowledge, this is the first report on population differences between Indians from two geographical regions in north and south India which additionally investigated whether differential positive selection in the two populations can explain the origins of the differences. This required population-level genome-wide SNP data to be available, as compared to previous reports of historical migration that relied primarily on chromosome Y and mitochondrial DNA data. Our discovery that the region around the *SLC24A5* skin pigmentation gene was positively selected in north Indians but not in south Indians may provide molecular evidence of sexual selection in the Indian society with its historical preference for fairer skin complexion. We envisage that further illuminating insights may be obtained with additional genome-wide SNP data across similar number of samples from other Indian populations or caste groups.

## Methods

### Datasets

Our analyses utilized the genome-wide genotype data from two sources. Phase 3 of the International HapMap Project surveyed 88 Gujarati Indians residing in Huston Texas (abbreviated GIH) across 1,389,511 autosomal SNPs [13], where three samples were subsequently excluded due to relatedness yielding a final sample size of 85 Gujarati Indians for analysis (release 3 of HapMap 3). The Singapore Genome Variation Project surveyed 83 unrelated Singapore Indians (abbreviated INS), where ethnic membership were ascertained by confirming that all four grandparents of each INS sample belonged to the Indian ethnic group. While it was not possible to ascertain precisely the origins of these 83 Singapore Indians, the south Asian Indian population in Singapore predominantly consists of descendants from immigrants from Dravidian-speaking states of Kerala, Karnataka and Tamil Nadu in south India [15]. The INS data consists of 1,583,455 autosomal SNPs.

### Quantifying allele frequency differences with F_ST_

To identify SNPs where the frequencies of the alleles differ significantly between GIH and INS, we calculated Wright’s F_ST_[16] for each of the 1,362,474 autosomal SNPs that are present in both the GIH and INS resources. We used the formula for two populations, given as

$$F_{\text{ST}}=\frac{{\left(P_{1}-P_{2}\right)}^{2}}{\left(P_{1}+P_{2}\right)\left(2-P_{1}-P_{2}\right)}$$

where *p*_1_ and *p*_2_ denote the allele frequencies of a specific allele at a SNP in GIH and INS respectively. In addition, we calculated the empirical p-value for each F_ST_ value by counting the proportion of SNPs out of 1,362,474 SNPs that displayed F_ST_ values that are at least as large as that observed. This empirical p-value is meant to indicate whether the observed F_ST_ value is significantly different from bulk of the SNPs in the rest of the genome. As we chose to discount individual SNPs that possess large F_ST_ values due to the possibility that such one-off differences are artefacts attributed to genotyping errors, we adopted a region-based approach and searched for contiguous stretches of the genome that carried an excess of SNPs with extreme F_ST_ values. Each chromosome was divided into non-overlapping segments of 100 kb, and a binomial test was performed for each segment to calculate whether the number of SNPs that were present with empirical p-values < 0.01 were higher than expected by chance. For assessing the robustness of the findings, a similar analysis was performed with a window size of 500 kb at an empirical p-value threshold of 0.001. The regions across all 22 autosomal chromosomes were subsequently pooled together and ranked, and regions found in the top 0.1% of the respective genome-wide distributions for the 100 kb and 500 kb analyses were considered to be significantly different between GIH and INS.

### Principal components analysis

We used the *pca* option that is available as part of the *eigenstrat* software [34] to perform principal components analyses (PCA). Three different PCAs were carried out: (i) with 1068 samples from INS and the 11 HapMap 3 populations across 1,362,474SNPs; (ii) with 85 GIH, 83 INS and 132 Indian samples from a population genetics survey of the Indian subcontinent by Reich and colleagues [1] across a total of 451,699 SNPs; and (iii) with only the 85 GIH and 83 INS samples across 1,362,474 SNPs. To avoid confounding the comparison due to the different number of SNPs and to reduce the impact of correlated SNPs, we thinned the set of 451,699 SNPs (from the second comparison) to 112,925 SNPs by selecting the first SNP out of every four consecutive SNPs as the placement of SNPs in the microarrays were predominantly chosen on their ability to tag surrounding SNPs. This set of SNPs is subsequently used in the three PCAs. The proportion of the variance explained by each principal component is calculated by the ratio of the corresponding eigenvalue to the sum of all eigenvalues.

### STRUCTURE analyses

We used the *STRUCTURE* program (version 2.3.4) to determine the level of admixture present in the GIH and INS samples. We used the following four populations from HapMap 3 as a baseline for calibration: (i) 112 Utah residents with northern and western European ancestry (CEU); (ii) 89 Toscans in Italy (TSI); (iii) 85 Han Chinese in Beijing, China (CHB); and (iv) 113 Yoruba from the Ibadan region of Nigeria (YRI). The analysis was performed with five different sets of 10,000 randomly selected SNPs across the genome. The admixture model was selected as the ancestral model that assumed the genome of each individual is a mosaic of the content from *K* populations, where the *K* parameter was set to 4, 5 and 6. No prior population information was provided in the analysis, and we run the analysis with a burn-in of 10,000 iterations and for 20,000 samplings, where the posterior mean estimates across the 20,000 samplings were used to calculate the admixture proportion from the *K* populations for each individual.

### Positive selection with iHS and XP-EHH

The iHS [17] and XP-EHH [18] metrics were used to locate differential genomic signatures of positive selection in GIH and INS. We used the C++ programs available at http://hgdp.uchicago.edu/Software/ for iHS and XP-EHH to perform the analyses [35] on the phased haplotypes that are publicly available from the HapMap and SGVP resources. The population-averaged recombination rates from Phase 2 of HapMap were used in the calculations. All iHS and XP-EHH analyses are performed on the set of 1,362,474 autosomal SNPs that are present in both GIH and INS to avoid any artifacts that may be caused by the difference in SNP densities between the two databases. For iHS, the raw statistics were normalized within each of the 20 derived allele frequency bins that spanned 5%. We identify genomic regions where the normalized iHS scores were present in the top 0.1% of the genome-wide distribution in one population but was not present in the top 1% of the genome-wide distribution in the other population. In order for a region to qualify as a differential selection signal, at least one SNP should be present in the top 0.1% of genome-wide distribution in one population, while there are no SNPs that are at the top 1% of the genome-wide distribution of the other population in that region. For XP-EHH, the analysis was performed with GIH and INS and the raw scores were normalized to have a zero mean and unit variance. We searched for clusters of SNPs with large absolute values for the normalized XP-EHH scores, which will indicate that a selection event is likely to have happened in one population but not in the other. The direction of each signal indicated whether the selection event happened in GIH (negative) or INS (positive). Regions with signals in the top 0.01% of either extreme of the genome-wide distribution of the XP-EHH scores were considered to exhibit differential evidence of positive selection.

### Calculating haplotype similarity

To evaluate the extent of similarity between GIH haplotypes and those from southern Europe (TSI) and south India (INS), we divided each chromosome into non-overlapping windows of 100 kb and calculate a haplotype similarity score between GIH and TSI, and between GIH and INS [25]. In each 100 kb window for a population pair, we identified the set of unique haplotypes that are present with frequencies of at least 2% in each population. The haplotype similarity score is defined as the proportion of the haplotypes across the two populations that have been represented by these unique haplotypes, and this is a metric bounded between 0 and 1 where larger values indicate there are greater haplotype sharing between the two populations.

## Competing interests

The authors declare that they have no competing interests.

## Authors’ contributions

YYT conceived, designed and directed the experiment; MA, XL. ENP and PC analzyed the data; YYT, CCK and RTHO wrote the paper. All authors read and approved the final manuscript.

## Supplementary Material

Additional file 1 Figures for rest of the significantly differentiated regions between INS and GIH. Click here for file
