# Supplementary material for: Characterizing the genetic differences between two distinct migrant groups from Indo-European and Dravidian speaking populations in India
Source: BMC Genet. 2014 Jul 22;15:86. doi: 10.1186/1471-2156-15-86 (PMC4120727; doi:10.1186/1471-2156-15-86)
Supplement: Additional file 1 — Figures for rest of the significantly differentiated regions between INS and GIH. [file 1471-2156-15-86-S1.pdf]

SUPPLEMENTARY MATERIAL

Characterizing the genetic differences between North and South Indians

Ali et al

CONTENTS

Figure S 1 2
Figure S 2 3
Figure S 3 4
Figure S 4 5
Figure S 5 6

Figure S1. Genetic differentiation between GIH and INS on chromosome 17
Evidence of genetic differentiation between GIH and INS on chromosome 17 between 41.3Mb and 41.5Mb from three discovery mechanisms that look for considerable regional differences in SNP allele frequencies (as quantified by the F_{ST} metric) relative to the genome (top panel); differential evidence of positive selection from iHS in GIH and INS (middle panel); XP-EHH signals contrasting GIH and INS that are found in either tails of the genome-wide distribution (bottom panel). In all three panels, SNPs exhibiting extreme evidence relative to the genome are displayed in differently in yellow, orange and red according to the respective percentiles as illustrated in the three figure legends. Genes located within this region are identified according to NCBI hg18 (build 36) coordinates.

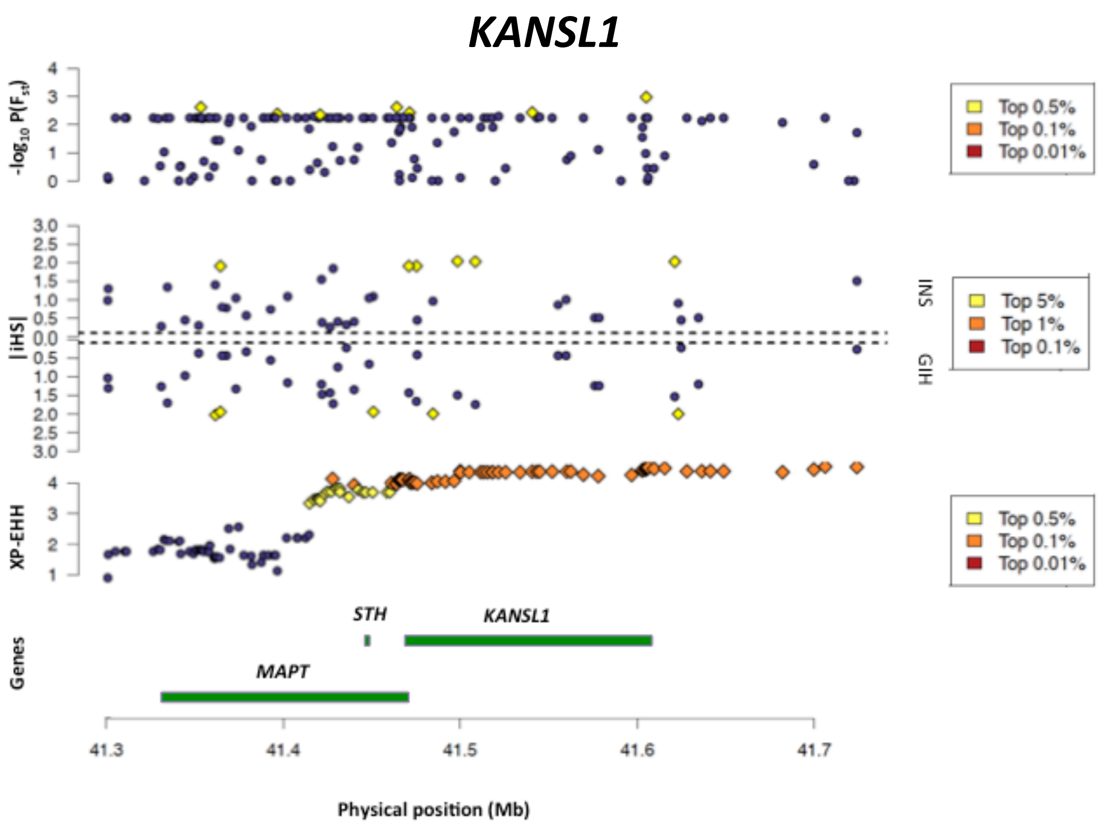

Figure S 1

Figure S2. Genetic differentiation between GIH and INS on chromosome 4
Evidence of genetic differentiation between GIH and INS on chromosome 4 between 17.0Mb and 17.5Mb from three discovery mechanisms that look for considerable regional differences in SNP allele frequencies (as quantified by the F_{ST} metric) relative to the genome (top panel); differential evidence of positive selection from iHS in GIH and INS (middle panel); XP-EHH signals contrasting GIH and INS that are found in either tails of the genome-wide distribution (bottom panel). In all three panels, SNPs exhibiting extreme evidence relative to the genome are displayed in differently in yellow, orange and red according to the respective percentiles as illustrated in the three figure legends. Genes located within this region are identified according to NCBI hg18 (build 36) coordinates.

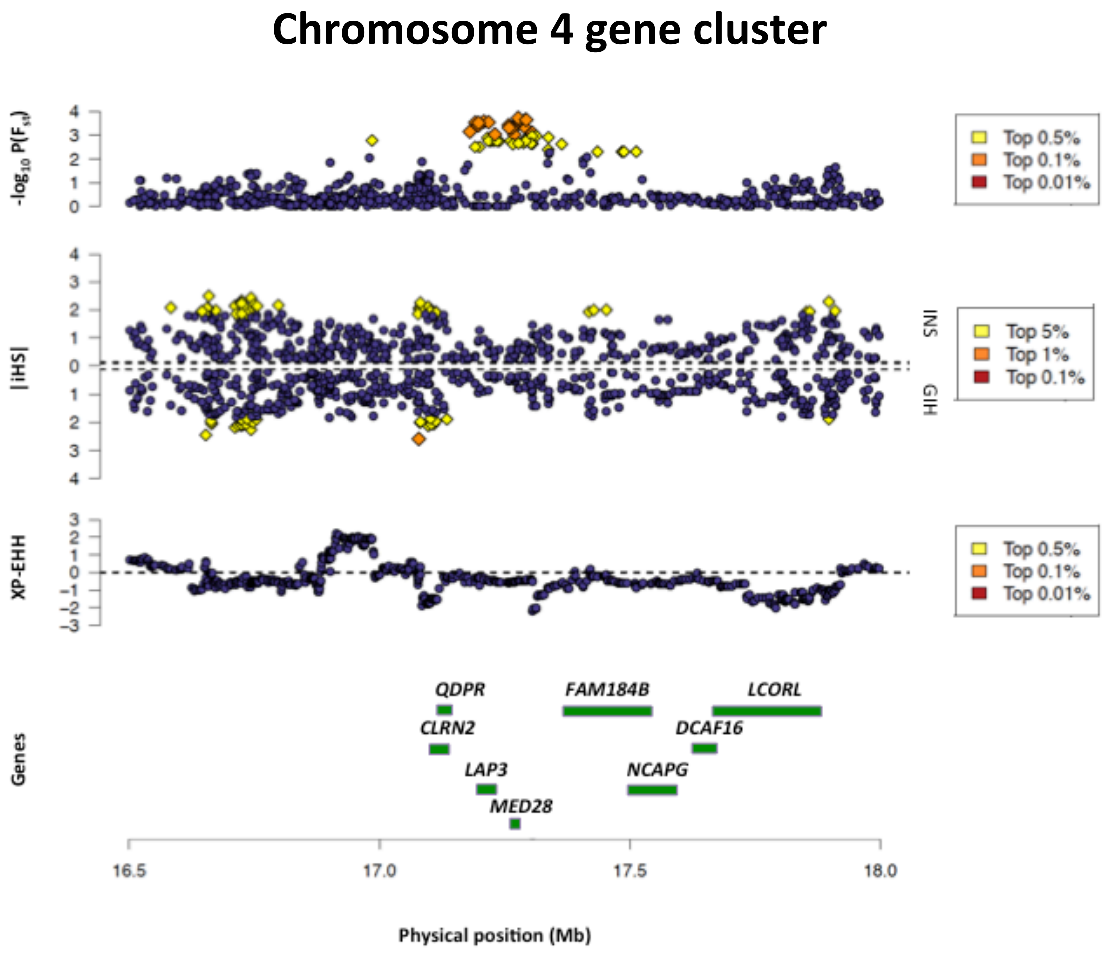

Figure S 2

Figure S3. Genetic differentiation between GIH and INS on chromosome 8
Evidence of genetic differentiation between GIH and INS on chromosome 8 between 85.0Mb and 86.0Mb from three discovery mechanisms that look for considerable regional differences in SNP allele frequencies (as quantified by the F_{ST} metric) relative to the genome (top panel); differential evidence of positive selection from iHS in GIH and INS (middle panel); XP-EHH signals contrasting GIH and INS that are found in either tails of the genome-wide distribution (bottom panel). In all three panels, SNPs exhibiting extreme evidence relative to the genome are displayed in differently in yellow, orange and red according to the respective percentiles as illustrated in the three figure legends. Genes located within this region are identified according to NCBI hg18 (build 36) coordinates.

RAYL

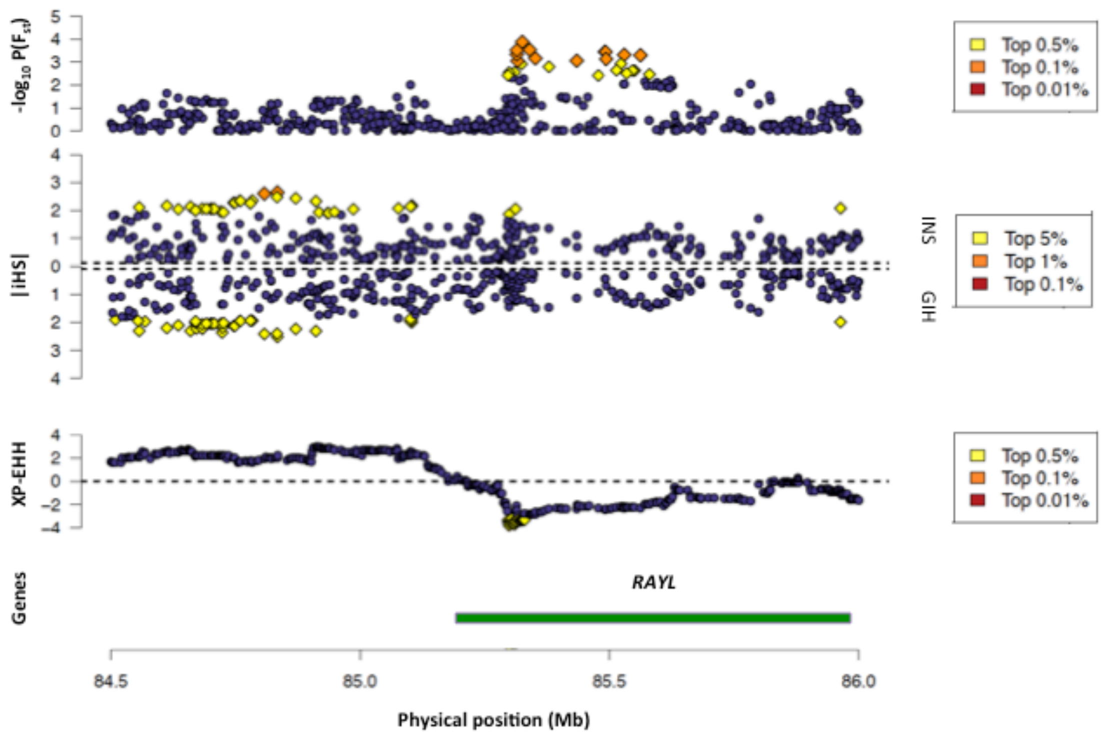

Figure S 3

Figure S4. Genetic differentiation between GIH and INS on chromosome 12
Evidence of genetic differentiation between GIH and INS on chromosome 12 between 58.3Mb and 58.6Mb from three discovery mechanisms that look for considerable regional differences in SNP allele frequencies (as quantified by the F_{ST} metric) relative to the genome (top panel); differential evidence of positive selection from iHS in GIH and INS (middle panel); XP-EHH signals contrasting GIH and INS that are found in either tails of the genome-wide distribution (bottom panel). In all three panels, SNPs exhibiting extreme evidence relative to the genome are displayed in differently in yellow, orange and red according to the respective percentiles as illustrated in the three figure legends. Genes located within this region are identified according to NCBI hg18 (build 36) coordinates.

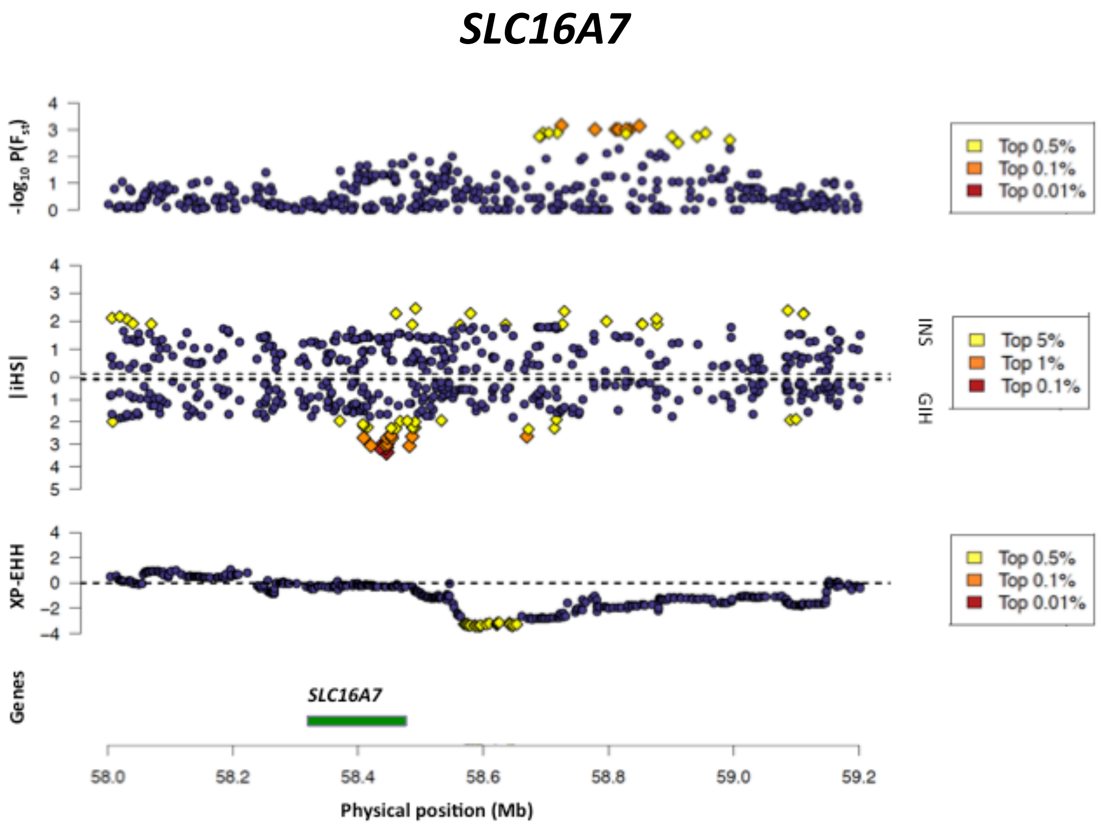

Figure S 4

Figure S5. Genetic differentiation between GIH and INS on chromosome 12
Evidence of genetic differentiation between GIH and INS on chromosome 12 between 80.3Mb and 80.6Mb from three discovery mechanisms that look for considerable regional differences in SNP allele frequencies (as quantified by the F_{ST} metric) relative to the genome (top panel); differential evidence of positive selection from iHS in GIH and INS (middle panel); XP-EHH signals contrasting GIH and INS that are found in either tails of the genome-wide distribution (bottom panel). In all three panels, SNPs exhibiting extreme evidence relative to the genome are displayed in differently in yellow, orange and red according to the respective percentiles as illustrated in the three figure legends. Genes located within this region are identified according to NCBI hg18 (build 36) coordinates.

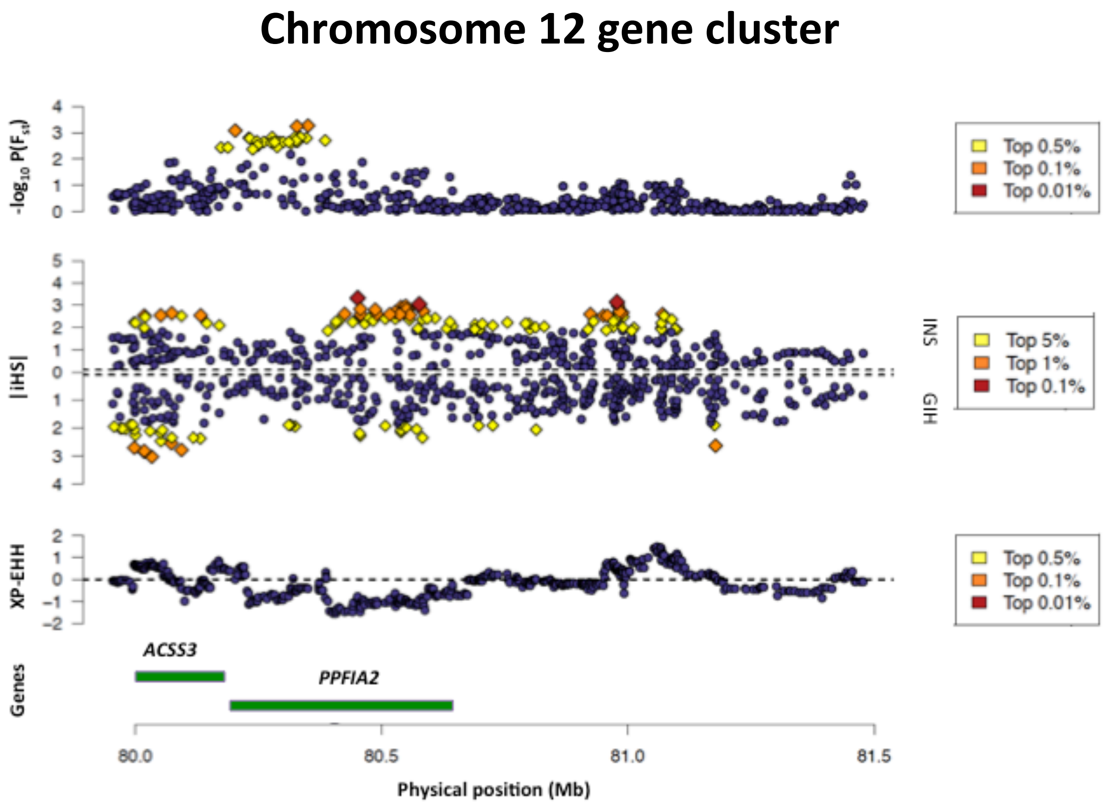

Figure S 5
